# Status and influential factors of health literacy in patients with ischemic stroke: A cross-sectional study

**DOI:** 10.1371/journal.pone.0307928

**Published:** 2024-07-29

**Authors:** Weixiu Ouyang, Rui Wang, Yueyue He, Yuqing Song, Linqi Mo, Ling Feng

**Affiliations:** 1 Department of Neurology, West China Hospital, Sichuan University/West China School of Nursing, Sichuan University, Chengdu, PR China; 2 School of Nursing, Chengdu University of Traditional Chinese Medicine, Chengdu, PR China; Chinese Academy of Medical Sciences and Peking Union Medical College, CHINA

## Abstract

**Aim:**

The purpose of this study was to investigate the health literacy level and influencing factors of patients with ischemic stroke.

**Design:**

A cross-sectional study was conducted.

**Methods:**

We used convenient sampling to recruit potential participants. Patients with ischemic stroke who were hospitalized in the Department of Neurology, West China Hospital, Sichuan University in China from January 2022 to May 2023 were selected as subjects to complete the questionnaire survey. We used the National Institute of Health Stroke Scale to assess the neurological impairment of patients and the Health Literacy Scale for Chronic Patients to assess the health literacy level of patients.

**Results:**

The overall health literacy level of ischemic stroke patients was high (99.13 ± 10.15). Age, education level, per capita monthly family income, living situations and National Institutes of Health Stroke Scale score were independent risk factors affecting the health literacy level of patients with ischemic stroke. Patients with a higher education level (*β* = 0.151, *P* = 0.006) and higher per capita monthly family income (*β* = 0.179, *P* = 0.001) had higher levels of health literacy. Patients who lived with their spouses (*β* = -0.117, *P* = 0.012) had higher health literacy. Patients at an older age (*β* = -0.151, *P* = 0.001) and patients with higher NIHSS scores (*β* = -0.450, *P* = 0.001) had lower health literacy levels.

**Conclusions:**

Age, education level, family per capita monthly income, living situations and National Institute of Health Stroke Scale score were related to the health literacy level of patients with ischemic stroke. According to these associated factors, targeted health education should be developed to improve the health literacy of patients and health outcomes.

## 1. Introduction

The 2019 Global Burden of Disease showed that stroke is the second leading cause of death and the third leading cause of disability in the world [1, 2]. The global cost of stroke is estimated over US$ 891 billion (1.12% of the global gross domestic product) [2, 3]. The “China Stroke Surveillance Report 2021” showed that a total of 3,418,432 stroke cases were admitted in China during 2020, over 80% (81.9%) were ischemic stroke (IS), and the medical cost of hospitalization for stroke in 2020 was CNY 58.0 billion, of which the patient pays approximately CNY 19.8 billion [4]. During the past three decades, in absolute terms, the global stroke incidence increased by 70%, its prevalence increased by 85%, and its mortality increased by 43%, with a greater increase in stroke burden in low-income and middle-income countries (LMICs) than in high-income countries [5]. Stroke is characterized by high morbidity, disability, mortality and recurrence and is the leading cause of death and disability among Chinese adults [6]. In a cross-sectional study of 676,394 participants aged 40 years and older, the estimated prevalence, incidence, and mortality rate of stroke in China in 2020 were 2.6%, 505.2 per 100 000 person-years, and 343.4 per 100 000 person-years [7]. Ischemic stroke patients in the United States account for 87% of all stroke patients [8]. In Sweden, approximately 22,000 people with a mean age of 75 years have a stroke each year, of whom 20% have a recurrent stroke [9]. the China Stroke Prevention Project Committee (CSPPC) investigated 12-month stroke fatality and recurrence rates after the first-ever stroke through a prospective nationwide hospital-based cohort study and the data showed that the 12-month fatality rate for ischemic stroke survivors was 6.0%, and the recurrence rate was 6.4% [4]. For people with ischemic stroke, the ability to understand and use health information is important to prevent recurrent strokes and to regain function [10].

Health literacy (HL) refers to the ability of individuals to obtain, understand and process basic health information and services and make correct health-related decisions [11]. At present, the level of health knowledge popularization among the masses is generally low. A European study found that 47% of the general population had limited (insufficient or problematic) health literacy [12]. Most citizens of the Czech Republic (58.5%) have sufficient health literacy [13]. A study on the level of health literacy of high-risk stroke groups in Jilin Province showed that only 18.03% of high-risk stroke groups had basic health literacy, and the overall level of health literacy was low [14]. A recent study on cardiovascular and cerebrovascular disease outpatient patients in the United Arab Emirates reported that 39.3% of respondents possessed inadequate HL [15]. In Taiwan, stroke history was reported in 4.3% of participants, and 25.3% reported low health literacy [2]. Previous studies have shown that the treatment compliance of stroke patients is related to their health literacy level [16], and low health literacy may increase the risk of recurrence [17]. The Healthy China Action Plan (2019–2030) [18] revealed that the burden caused by chronic diseases such as cardiovascular and cerebrovascular diseases accounted for more than 70% of the total disease burden, and the health literacy level of residents was only 14.18% in 2017. Improving health literacy should be taken as the basic way to improve the health of all people to improve their health level. Among people with cardiovascular and cerebrovascular diseases, significant associations were found between higher health literacy and higher levels of physical activity, healthier diet, and better self-reported health outcomes [19]. A study in patients with heart failure showed that low education level and household income level predicted poor health literacy [20]. Limited health literacy prevents individuals and families from developing the knowledge, skills, and confidence necessary to engage or participate in their care [21]. Although there have been studies on the status quo of health literacy in patients with stroke, there are few studies on the status quo and influencing factors of health literacy in patients with ischemic stroke. This study aimed to investigate the status quo and influencing factors of health literacy in patients with ischemic stroke.

## 2. Materials and methods

### 2.1. Participants

This study was a cross-sectional survey. Patients with ischemic stroke who were hospitalized in the Department of Neurology, West China Hospital, Sichuan University in China from January 2022 to May 2023 were selected as subjects using a convenience sampling method. The inclusion criteria were as follows: ① Diagnosed as having an ischemic stroke by a neurologist through CT and MRI; ②The patient was ≥18 years old; ③There was no cognitive impairment, no communication impairment; ④Vital signs are stable, and the patient could cooperate; and ⑤The patient volunteered to participate in this study and signed the consent form. The exclusion criteria were as follows: ① serious mental disorders; ② obvious dysfunction of the heart, liver and lung; ③ other serious complications; and ④ inability to cooperate with the questionnaire survey or inability to complete the questionnaire. This study was approved by the Ethics Committee of West China Hospital, Sichuan University in China number2019(728).

### 2.2. Instruments

#### 2.2.1. General characteristics questionnaire

We designed this questionnaire to assess the general characteristics of the patients. It mainly includes the patient’s age, gender, education level, occupation, family per capita monthly income, medical payment method, whether the patient has chronic diseases such as hypertension, whether the patient takes aspirin or clopidogrel and other antiplatelet agglutination drugs, and whether the patient takes lipid-lowering drugs.

#### 2.2.2. National Institutes of Health Stroke Scale (NIHSS)

The National Institutes of Health Stroke Scale (NIHSS) is a 15-item impairment scale used to measure stroke severity [22]. The total score ranges from 0 to 42, and the higher the score is, the more serious the nervous system function injury [23]. The NIHSS has moderate-to-high reliability when carried out by medical and nonmedical staff (intrarater *κ* = 0.66 to 0.77; interrater *κ* = 0.69). Very high reliability has also been demonstrated when clinicians rate from videos of patients (intrarater ICC = 0.93; interrater ICC = 0.95) [22]. According to the Stroke Scale score of the National Institutes of Health, NIHSS > 14 was classified as severe stroke patients, and NIHSS≤14 was classified as mild to moderate stroke patients [24, 25].

#### 2.2.3. Health literacy scale

The Health Literacy Scale for Chronic Patients (HLSCP) is a scale used to evaluate the health literacy level of patients with chronic diseases, and it was revised from the Health Literacy Management Scale [26, 27]. The scale has a total of 24 items, including the ability to obtain health information, the ability to communicate and interact, the willingness to improve health, and economic support. The Likert 5-level scoring method was adopted for each item, with a total score of 120. The higher the score, the better the health literacy. Cronbach’s α coefficient of the scale was 0.894, and the retest reliability was 0.683. When the score or total score of each dimension exceeds 80%, the patient’s health literacy level is considered to be good; that is, when the total score of health literacy is ≥96, the patient’s health literacy level is good.

### 2.3. Data Collection

**Fig 1:**
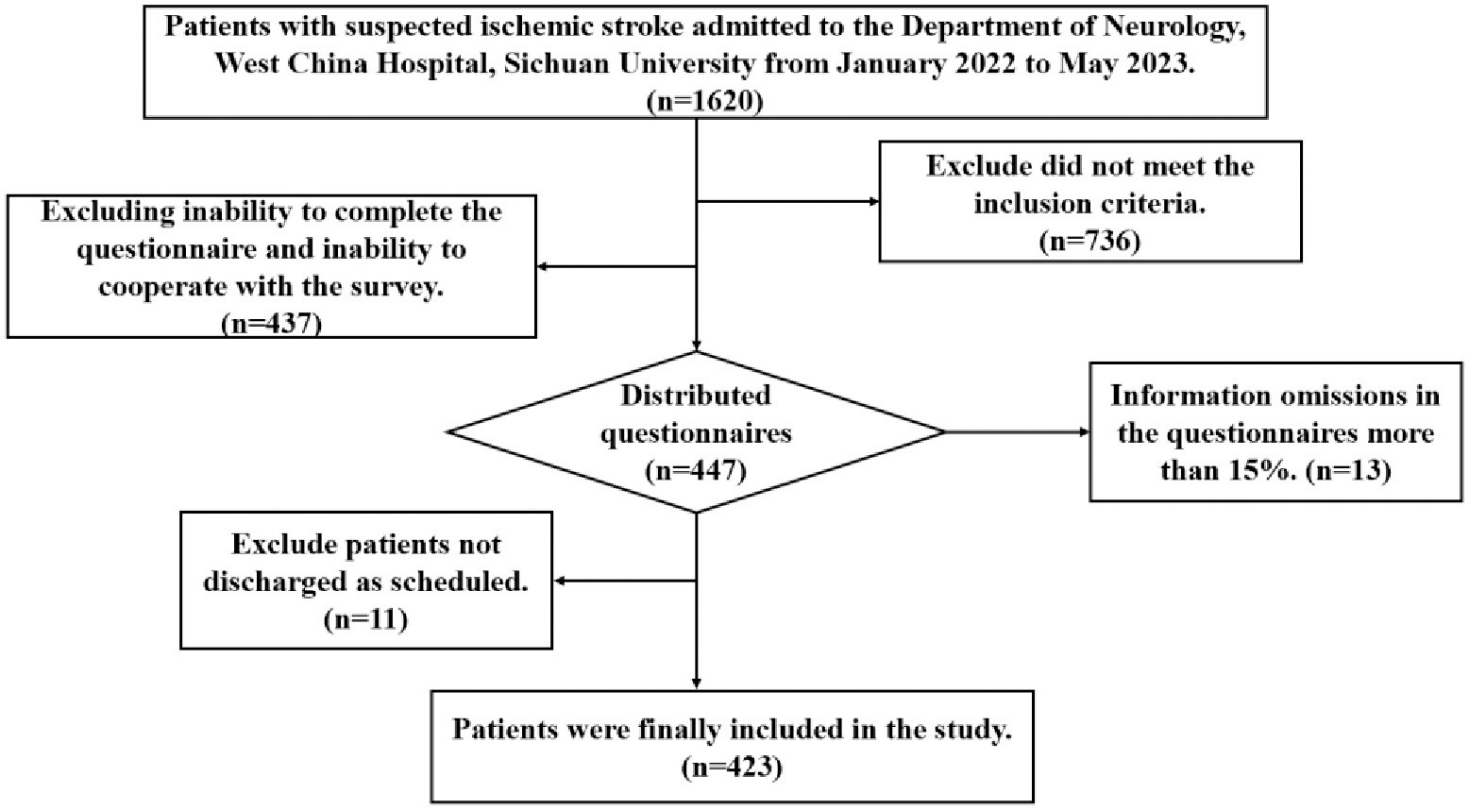
Flowchart of the study population.

The patients completed the general demographic data questionnaire and health literacy survey of chronic disease patients by themselves. The researchers explained the purpose and significance of the study to the patients and their families and conducted a one-to-one questionnaire survey after obtaining the informed consent of the patients. For the patients who could not complete the questionnaire due to vision decline or illiteracy, the researchers assisted them. The questionnaire was completed according to the patient’s answers to ensure the completeness of the questionnaire. The National Institutes of Health Stroke Inventory was obtained by reviewing patient records. A total of 447 questionnaires were sent out in this study. After excluding invalid questionnaires, 423 valid questionnaires were collected (Fig 1), with an effective recovery rate of 94.6%. The average filling time was 10–20 minutes.

### 2.4. Statistical analysis

Descriptive statistics and frequencies were carried out to describe the sociodemographic and health literacy scores. The scores of the HLSCP were tested for normality and are shown as the mean and standard deviation. The correlation between the NIHSS scores and the level of health literacy was calculated using the Spearman correlation coefficient. Before developing the multiple linear regression model, we checked the linear relationship between the independent variables and the dependent variables, the distribution of residuals, the variance of the residuals, the independence between the residuals, and the collinearity between independent variables. Meanwhile, multicollinearity, outliers and leverage points were also tested. Then, the forward stepwise regression method was used to select the regression variables, and the multiple linear regression model was developed after screening the meaningful indicators. Only variables with *P*<0.05 in the correlation analysis were included. SPSS 26.0 software was used for statistical analysis. Statistical tests were two-tailed, and statistical significance was set at 0.05.

## 3. Results

A total of 423 patients participated in the survey, of which 298 (70.4%) were male. There were 260 patients (61.5%) aged 60 years and above. The number of patients with a high school education or below was 312 (73.8%), and the number of patients with a bachelor’s degree or above was 47 (11.1%). The number of retired patients was 185 (43.7%). The number of spouses who were caregivers for patients was 216 (51.1%). There were 150 patients (35.5%) whose per capita family income was 5,000 yuan or above. Patients with other chronic diseases accounted for 80.1%. Most of the patients (74%) had urban employee medical insurance or urban resident medical insurance. The vast majority of patients (74.2%) lived with their partner (Table 1).

**Table 1:** Univariate analysis of factors influencing health literacy in patients with ischemic stroke.

Characteristics		n (%)	total health literacy score	t/F	*p* value
gender	male	298 (70.4)	99.87±17.29	t = 1.292	0.197
	female	125 (29.6)	97.38±20.02		
age	18–44 years old	26 (6.1)	110.50±12.91	F = 8.564	<0.001
	45–59 years old	137 (32.4)	101.38±15.98		
	age 60 and above	260 (61.5)	96.82±19.12		
degree of education	primary school and below	147 (34.8)	93.02±18.22	F = 10.222	<0.001
	junior high school	115 (27.2)	98.78±16.26		
	senior high school	50 (11.8)	105.40±14.88		
	technical secondary school	26 (6.1)	94.92±28.06		
	junior college	38 (9)	104.21±13.85		
	Bachelor’s degree or above	47 (11.1)	110.68±12.85		
occupational status	active personnel with stable income	49 (11.6)	107.59±13.13	F = 5.688	<0.001
	retirement	185 (43.7)	99.95±18.47		
	farmer	77 (18.2)	93.97±17.99		
	unemployed	60 (14.2)	94.53±16.79		
	others (personnel with no stable income)	52 (12.3)	101.23±19.59		
caregiver	spouse	216 (51.1)	101.58±16.56	F = 4.475	0.001
	children	162 (38.3)	95.13±18.95		
	parents	8 (1.9)	114.25±8.53		
	care worker/nanny	18 (4.3)	95.39±21.93		
	unaccompanied	8 (1.9)	110.63±7.86		
	others	11 (2.6)	96.91±24.51		
per capita monthly income of households	≤2000yuan	18 (4.3)	82.06±24.92	F = 9.740	<0.001
	2000–3000yuan	37 (8.7)	90.86±17.06		
	3000–4000yuan	109 (25.8)	96.40±16.71		
	4000–5000yuan	109 (25.8)	102.12±12.61		
	≥5000yuan	150 (35.5)	103.04±19.87		
is it complicated with chronic diseases	yes	339 (80.1)	98.51±18.69	t = -1.43	0.153
	no	84 (19.9)	101.67±15.64		
medical insurance type	urban employee medical insurance	190 (44.9)	101.58±19.22	F = 8.699	<0.001
	urban resident medical insurance	123 (29.1)	101.59±14.70		
	the new rural cooperative medical insurance	101 (23.9)	91.30±18.44		
	others	9 (2.1)	101.78±9.18		
smoking history	yes	203 (48)	99.10±17.96	t = -0.34	0.973
	no	220 (52)	99.16±18.37		
drinking history	yes	183 (43.3)	97.72±19.09	t = -1.4	0.162
	no	240 (56.7)	100.21±17.36		
Living situations	living alone	22 (5.2)	97.45±24.10	F = 13.704	<0.001
	living with a spouse	314 (74.2)	101.64±15.92		
	others	87 (20.6)	90.51±21.29		

The results of the univariate analysis of influencing factors of health literacy in patients with ischemic stroke are shown in Table 1. Through the comparison of different demographic characteristics of patients, the univariate analysis showed that the health literacy levels of patients with ischemic stroke were different with different ages, educational levels, occupational statuses, caregivers, per capita monthly family income, types of medical insurance and living situations, and the differences were statistically significant (*P*<0.05).

The health literacy scores of patients with ischemic stroke are shown in Table 2. The total health literacy score was 99.13 ± 10.15. The average score of the 423 patients with ischemic stroke included in the study was 31.31±7.03 in the dimension of information acquisition ability and 41.09±8.30 in the dimension of communication and interaction ability. The average score of the improvement intention dimension was 17.87±3.27 points, and the average score of economic support intention was 8.86±1.35 points. The health literacy level of the patients with ischemic stroke in this study was 68.79%.

**Table 2:** Scores of all dimensions of health literacy in patients with acute ischemic stroke.

Variables	Minimum value	Maximum value	Mean value	Standard deviation	Scoring rate
Information acquisition ability dimension score	8	40	31.31	7.03	78.28%
Score on the dimension of communication and interaction	10	50	41.09	8.30	82.18%
Improved health willingness dimension score	4	20	17.87	3.27	89.35%
Economic support dimension score	2	10	8.86	1.35	88.60%
Overall health literacy score	30	120	99.13	10.15	82.61%

The multifactor analysis of influencing factors of health literacy in patients with ischemic stroke is shown in Table 3. Health literacy in patients with ischemic stroke was generally divided into dependent variables, and significant correlation variables in the univariate analysis were taken as independent variables. Age, education, occupational status, caregiver, monthly income per capita in the family, type of health insurance, living situations and NIHSS scores were included as independent variables. Through multiple stepwise linear regression analysis, the results showed that age, education level, per capita monthly family income, living situations and NIHSS scores were the factors influencing the health literacy level of patients with ischemic stroke (*P*<0.05).

**Table 3:** Multiple stepwise linear regression analysis of influencing factors on health literacy level in patients with acute ischemic stroke.

Variables	Unstandardized coefficient		Standardization coefficient	t	*P* value
	B	Standard error	Beta		
(constant)	100.893	5.922		17.038	<0.001
Degree of education	1.574	0.568	0.151	2.771	0.006
Per capita monthly household income	2.798	0.848	0.179	3.298	0.001
age	-4.509	1.384	-0.151	-3.259	0.001
Living situations	-3.549	1.413	-0.117	-2.512	0.012
NIHSS score	-2.909	0.263	-0.450	-11.051	0.001

R^2^ = 0.327, adjusted R^2^ = 0.320, F = 17.780

## 4. Discussion

This study investigated the health literacy level of patients with ischemic stroke during hospitalization and discussed the influencing factors of health literacy. The results showed that 68.79% of patients with ischemic stroke had a good level of health literacy. Results of a Swedish study investigated health literacy levels in stroke patients 12 months after discharge showed that 62% of participants had adequate health literacy [28]. This was consistent with our findings, possibly because stroke patients received health care services both during hospitalization and after discharge, and received stroke-related health education, thereby learned more stroke-related health knowledge. The results of our study showed that participants’ willingness to improve their health scored the highest, indicating that participants were eager to recover their health after stroke and were willing to accept health education with a more positive attitude, so as to have a good level of health literacy. However in another study, only 18.03% of people at high risk for stroke had basic health literacy, indicating an overall low level [14]. It was different from the results of our study, on the one hand, it may be because the participants’ basic knowledge level was low, the understanding of stroke was little, and the participants were mainly 45–69 years old, but also the life and career of the high risk group, so their health problems were often ignored [14]. On the other hand, it may be because health knowledge about stroke prevention and treatment involved multiple specialties and was not widespread enough in daily life. Previous research has shown that health literacy can be spread and improved through interactions with social networks and healthcare professionals [29]. Therefore, we can carry out health education for different groups through more diversified popular science methods to improve their health literacy level.

The multiple stepwise linear regression analysis showed that degree of education, age, per capita monthly income, living situations and NIHSS scores were the main factors affecting ischemic stroke patients. In this study, the higher the education level of patients with ischemic stroke was, the higher the level of health literacy, which is basically consistent with the results of previous studies [30–32]. Education was the basis of health literacy, and previous studies had identified the link between education levels and health literacy; well-educated elderly patients had better learning and understanding abilities [33]. It may be that patients with higher education levels have a higher acceptance of health education and can take the initiative to obtain health information in the process of health promotion, thus having a higher level of health literacy. Lower education was associated with lower health literacy [34]. Results from a multicenter study that explored the relationship between patient health literacy and disease perception and health-related quality mentioned that participants in eight out of 12 countries reported that the most common difficulty affecting health literacy was "reading and understanding all the information on pharmaceutical labels" [35]. Patients with lower education levels have limited access to information due to limitations in reading and comprehension abilities, making it difficult to ensure the effectiveness of health education. This also suggests that we should focus on strengthening health education for people with lower educational levels in a more understandable way to improve the overall health literacy level of patients.

Age was one of the factors affecting the health literacy level of patients with ischemic stroke. Older age was associated with lower levels of health literacy, which was consistent with previous research [36]. Compared with patients aged between 18 and 44 years old, the health literacy level of elderly patients over 60 years old was lower [27]. The possible reason is that with age, body function gradually decreases. The elderly’s ability to accept, understand, remember and learn new things was reduced, leading to a low level of health literacy [37, 38]. The majority of stroke patients are middle-aged and elderly, and their ability to understand and receive health information is lower than that of young people. Therefore, when conducting health education for patients with ischemic stroke, the frequency of health education should be appropriately increased according to the age and specific conditions of patients, so as to help patients master stroke-related health knowledge and improve their health literacy.

In this study, patients with a per capita monthly family income of 3000 yuan and above had better health literacy. The level of health literacy of patients with ischemic stroke was also affected by the level of per capita monthly family income. This result is consistent with the research results of Lin and Xiao [39] and Schaeffer et al. [32]. Patients with higher income had higher health literacy scores, and patients were more willing to improve their health status after meeting their daily basic needs. They tend to invest more in their own health management, which may account for their higher level of health literacy [30, 40]. Patients with less income have little time and not much extra money to take care of their health to guarantee material life, which results in patients having less access to health information [30, 41]. The World Health Organization stresses that improving health literacy is a public health goal [14]. Therefore, in order to ensure the effect of health education, personalized health education plans should be formulated according to the different ways of receiving health information for different income groups. Through diversified health education methods to improve their health literacy level.

In this study, living situation was also one of the factors affecting the health literacy level of patients with ischemic stroke. Living with a spouse was associated with higher levels of health literacy than living with other people. Research by Shi, Y. et al [36] showed that marital status and family structure played an important role in maintaining and promoting health literacy. Zhao, C. S. et al [14].found that there was a positive correlation between family function and health literacy level. After stroke, the self-care ability of patients decreased more or less, and the dependence on family and spouse increased. Therefore, when medical personnel carry out health education, the education object should include not only the patient himself but also the spouse of the patient. By improving the health literacy of family members and improving family function, the goal of improving the health literacy level of patients can be achieved [42].

The NIHSS can comprehensively assess the dysfunction of patients after stroke. The higher the NIHSS scores, the more serious the neurological impairment. Patients with high NIHSS scores usually have physical dysfunction [43]. Few previous studies have explored the relationship between NIHSS scores and health literacy in stroke patients. In this study, the higher the NIHSS scores of patients, the lower the level of health literacy. A previous study showed that health education can reduce the degree of neurological impairment in patients [44]. Results of a study examining the relationship between health literacy and reduced depressive symptoms, improved perceived recovery, improved perceived engagement, and the ability to walk one year after stroke showed that health literacy was associated with post-stroke outcomes [28]. So we can implement personalized health education for patients with different degrees of neurological impairment to improve their health literacy, so as to achieve the purpose of improving the prognosis.

## 5. Limitations

There are some limitations to the study. A stratified random sampling method was not adopted in this study to select survey objects, and all the subjects were from one hospital, so the representativeness of survey objects may be affected to some extent. In future studies, the sample size and distribution range should be expanded as much as possible. In addition, this study only investigated the health literacy level of patients during hospitalization. But health literacy is dynamically changing, so future studies should continue to follow up the health literacy level of patients after discharge to further explore the relationship between health literacy and stroke prognosis.

## 6. Conclusion

An important finding of this study is that there is a negative correlation between NIHSS scores and health literacy levels. This study showed that the health literacy level of patients with ischemic stroke was good. Age, education level, per capita monthly family income, living conditions and NIHSS scores were all influencing factors. It is also necessary to propose targeted health education according to the influencing factors to improve the health literacy of patients and improve their health outcomes.

## Supporting information

S1 Data (XLSX)
